# Discovering associations between problem list and practice setting

**DOI:** 10.1186/s12911-019-0779-y

**Published:** 2019-04-04

**Authors:** Liwei Wang, Yanshan Wang, Feichen Shen, Majid Rastegar-Mojarad, Hongfang Liu

**Affiliations:** Division of Digital Health Sciences, Department of Health Sciences Research, Mayo Clinic, Rochester, MN 55905 USA

**Keywords:** Problem list, Practice setting, Topic modeling, Statistical χ^2^ test, TF-IDF and enrichment analysis

## Abstract

**Background:**

The Health Information Technology for Economic and Clinical Health Act (HITECH) has greatly accelerated the adoption of electronic health records (EHRs) with the promise of better clinical decisions and patients’ outcomes. One of the core criteria for “Meaningful Use” of EHRs is to have a problem list that shows the most important health problems faced by a patient. The implementation of problem lists in EHRs has a potential to help practitioners to provide customized care to patients. However, it remains an open question on how to leverage problem lists in different practice settings to provide tailored care, of which the bottleneck lies in the associations between problem list and practice setting.

**Methods:**

In this study, using sampled clinical documents associated with a cohort of patients who received their primary care at Mayo Clinic, we investigated the associations between problem list and practice setting through natural language processing (NLP) and topic modeling techniques. Specifically, after practice settings and problem lists were normalized, statistical χ^2^ test, term frequency-inverse document frequency (TF-IDF) and enrichment analysis were used to choose representative concepts for each setting. Then Latent Dirichlet Allocations (LDA) were used to train topic models and predict potential practice settings using similarity metrics based on the problem concepts representative of practice settings. Evaluation was conducted through 5-fold cross validation and Recall@k, Precision@k and F1@k were calculated.

**Results:**

Our method can generate prioritized and meaningful problem lists corresponding to specific practice settings. For practice setting prediction, recall increases from 0.719 (k = 2) to 0.931 (k = 10), precision increases from 0.882 (k = 2) to 0.931 (k = 10) and F1 increases from 0.790 (k = 2) to 0.931 (k = 10).

**Conclusion:**

To our best knowledge, our study is the first attempting to discover the association between the problem lists and hospital practice settings. In the future, we plan to investigate how to provide more tailored care by utilizing the association between problem list and practice setting revealed in this study.

**Electronic supplementary material:**

The online version of this article (10.1186/s12911-019-0779-y) contains supplementary material, which is available to authorized users.

## Background

Since its enactment in 2009, the Health Information Technology for Economic and Clinical Health Act (HITECH) has greatly accelerated the adoption of electronic health records (EHRs) with the promise of better clinical decisions and patients’ outcomes. According to the Centers for Medicare & Medicaid Services (CMS), “meaningful use” of EHRs refers to the use of EHRs to achieve significant improvements in care. One of the core criteria for “Meaningful Use” of EHRs is to have a codified up to date problem list that lists the most important health problems faced by a patient [1–4]. The problem list was first introduced by Weed in 1968 in his promotion for a Problem-Oriented Medical Record (POMR) [5]. Since then it has been widely used and become a key component in patient records. In the Health Level Seven International’s Electronic Health Record System Functional Model (EHR-S FM), a problem list “may include, but is not limited to chronic conditions, diagnoses, or symptoms, functional limitations, visit or stay-specific conditions, diagnoses, or symptoms” [6].

Ideally, physicians could benefit from an accurate problem list to track a patient’s status and progress, to maintain continuity of patient care and to organize clinical reasoning and documentation [7]. Accurate problem lists could also be used for the improvement of the quality of care, the realization of clinical decision support, and the facilitation of research and quality measurement [8]. The problem list can serve a variety of uses in diverse healthcare settings by providing a succinct view of a patient’s health status and therefore should be used and maintained to meet different needs. For example, a primary care physician concerns chronic and acute conditions while a specialty provider may focus only on a subset of problems relevant to that area of medicine. An emergency provider may address only the critical acute presenting problems. Other clinicians may use the problem list for tracking conditions that should be addressed for specific care delivery goals. Extensive studies have been conducted to assess the usefulness of problem lists, for example, through the exploration of the use pattern of problem lists [9], the detection of problem list gaps in recording patients’ problems [10, 11], the creation and maintenance of a problem list using natural language processing [12–14], and the use of problem list for decision making support [15]. However, due to the inconsistent use across providers as well as the lack of the consensus of what should be documented in the problem lists [16], problem lists are frequently inaccurate and out-of-date [15]. It remains an open question how to leverage the problem list to provide tailored care at different practice settings (e.g., primary care, cardiology, or emergency) and for different care providers (e.g., clinicians, nurses, or social workers), of which the bottleneck lies in the associations between problem list and practice setting.

In this study, we aim to investigate the associations between the problem list and practice settings using the longitudinal EHR data from Mayo Clinic by mapping problems and practice settings to standard representations and assessing the associations between them using topic modeling [17] and clustered imaging map (CIM) [18].

## Methods

**Fig. 1:**
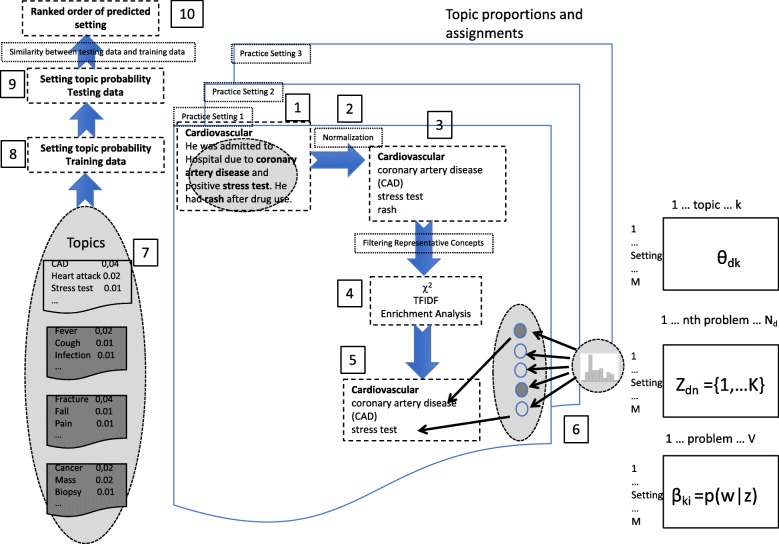
The overall workflow of the proposed method

Figure 1 illustrates the overall workflow in this study. Our method used natural language processing (NLP) to normalize problem list and manually aggregated practice settings (step 1–3), where “Cardiovascular” is the practice setting of the problems like “coronary artery disease”. Representative concepts were then filtered using χ2, term frequency-inverse document frequency (TF-IDF) and enrichment analysis based on the Semantic Medline (step 4–5). Subsequently Latent Dirichlet Allocations (LDA) [19] were used to train topic models and predict potential practice settings using similarity metrics based on the problem list (step 6–10). Finally 5-fold cross validation was utilized for evaluation, while cluster image map [20] revealing setting similarity from all randomly chosen data was used for visualization.

## Data sources

The collection of clinical documents used in our analysis consists of clinical notes for a cohort of patients receiving their primary care at Mayo Clinic, spanning a period of 15 years (1998–2013), and covering both inpatient and outpatient settings. Problems in those documents are generally itemized entries as either phrases (e.g., “*Allergic rhinitis/vasomotor rhinitis”*) or short sentences (e.g, “*Her asthma appeared to be very mild”*). After normalization of settings and problem list, we randomly selected 1000 notes (documents) for each of 64 settings as the input for filtering, in total 64,250 notes was used as input for the step 4 to choose representative concepts. Then 60,345 notes were kept for training topic model in step 6. We then randomly selected 200 notes from each setting as testing data, in total 13,498 notes was used as input for step 9 to test the predicted settings.

The latest version of Semantic Medline Database (SemMedDB) has more than 84.6 million semantic associations from 25,582,462 Medline citations up to Dec 312,015 from 1865, based on the natural language processing tool SemRep and Unified Medical Language System (UMLS) [21]. Among eight tables, the most comprehensive PREDICATION_AGGREGATE (PA) table contains all available information from the SemMedDB, including subject concepts, object concepts, sentence ID, PubMed IDs (PMIDs), and so on. Article level co-occurrences among subject-object concepts, i.e., 1,164,352 total co-occurrences of concepts from all practice settings were used in enrichment analysis for statistically significant concepts associated with each setting extracted from clinical notes in the SemMedDB.

### Normalization of settings

As a large volume of clinical documents has been generated in the context of EHRs, the HL7/LOINC Document Ontology (DO) was developed to support a range of use cases (e.g., retrieval, organization, display, and exchange) [22]. It contains a hierarchical structure comprising five axes: Kind of Document (KOD), Type of Service (TOS), Setting, Subject Matter Domain (SMD) and Role. Each axis contains a set of values. Some studies explored the applicability of DO in document representation and mapping [23, 24], and use of LOINC codes for document exchange in the clinical scenario [25, 26]. Other studies have focused on the improvement of axes of SMD [27], TOS [28], and Setting [29], mainly through increasing the coverage of each axis to make it more comprehensively representative. For example, Rajamani et al. proposed extended values for Settings of Care from 20 to 274, that fall into 14 main classes, such as Inpatient, Outpatient, Public Health, Community, and Mobile [29]. Currently the settings in Mayo clinic notes are relatively refined. First, locations are usually used for differentiating settings of the same practice (e.g., Family Medicine BA, Family Medicine KA, where BA and KA indicated locations). Second, more detailed classifications have been generated under specific specialties (e.g., “Ped Neonatology-I” and “Psych Ped SMH”, (SMH is a location of Mayo Clinic)). In this way, names of settings could provide plentiful information on subjects, specialties and locations. Such refinement could facilitate targeted treatment. However, it results in a large number of settings, e.g., during the study period, there are more than 1000 settings in clinical notes. This brings hurdles for the meaningful use of problem lists in different settings.

In this paper, we studied the settings associated with more than 4500 clinical notes based on proposed extended values for Settings of Care [29] for setting aggregation. Two steps were taken to aggregate various settings into more general ones. First, for practice settings with the same practice and various locations, we kept the subject and removed locations. For example, “Family Medicine BA” and “Family Medicine KA” were merged into “Family Medicine”. Second, for those settings with similar specialties, we aggregated them into the general settings. For example, “Ped Neonatology-I” and “Psych Ped SMH” were aggregated into “Pediatrics”. In total, 64 settings were aggregated corresponding to 266 practice settings.

### Normalization of problem list

With a good coverage of frequently used terms in problem lists [30], the CORE Problem List Subset has been created to align with the meaningful use requirement and better implement Systematized Nomenclature of Medicine Clinical Terms (SNOMED CT) in electronic health records (EHR) [30]_._ In a previous study [31], we assessed the coverage of SNOMED CT for codifying problem lists in narrative format by extracting itemized entries from clinical notes and normalize them to the Unified Medical Language System (UMLS) [32] concepts. In this study, we applied the same methodology but kept UMLS concepts that can be mapped to the CORE Problem List Subset codes (the August 2015 version of The CORE Problem List Subset of SNOMED CT was used). Only diagnosis related sections were kept for further study, e.g., “History of Present Illness” and “Diagnosis”.

### Filtering representative concepts for each setting

In order to choose representative concepts among randomly selected notes for each setting, first statistical χ^2^ test was conducted, then TF-IDF and enrichment analysis for co-occurring concepts in each setting performed based on Semantic Medline. The purpose of χ^2^ test is to find concepts having significant association with practice settings. TF-IDF helps to remove concepts that appear in most practice settings and can’t demonstrate their unique value for specific practice setting. In enrichment analysis, we used an external data source, Semantic Medline to verify if the concepts in each setting after χ^2^ and TF-IDF filtering were overrepresented in the large-scale Semantic Medline. More details will be discussed in the following paragraphs.

After NLP and setting aggregation, each document had a corresponding setting and contains a list of normalized Concept Unique Identifiers (CUIs) for problems. In our pilot experiment, we randomly selected 1000 notes (documents) for each of 64 settings for 5 times. We found that out of total 4573 normalized problems, around 3630 are covered by randomly selected notes, account for 79.4%. We can infer from these results that 1000 notes (documents) could represent the corresponding practice setting.

Therefore randomly selected 64,250 training documents were used as input for calculation of χ^2^, deriving χ^2^ values for 240,110 concept and practice setting pairs. After choosing those cocept and pracice setting pairs with χ^2^ > 6.64 (*P* < 0.01), 17,180 were kept. TF-IDF for these pairs was calculated using the Eq. 1.

1 $$ \mathrm{TFIDF}=\frac{\mathrm{Fc},\mathrm{s}}{\log \left(1+\mathrm{N}/\mathrm{nc}\right)} $$

where *Fc,s* denotes the frequency of the concept c in the setting s, *N* the total number of settings, and *nc* the number of settings containing the concept. Fourteen thousand, one hundred and sixty concept and practice setting pairs with TF-IDF greater than 1 were kept for further enrichment analysis.

Enrichment analysis, primarily based on Gene Ontology, has been used for summarizing and profiling a gene set [33]. Recently, a few studies explored different sources, i.e., the Medical Subject Headings for enrichment analysis [34, 35]. As one of repositories for semantic predications processed from the Medline, Semantic Medline has been employed for the discovery of relationships among biological entities [36]. In this study, we proposed to leverage the abundant entities and semantic associations in the Semantic Medline for concept co-occurring enrichment analysis to verify if the concepts in each setting after χ^2^ and TF-IDF filtering were overrepresented in the large-scale Semantic Medline.

Specifically we calculated the enrichment fold of concept-setting pairs. Enrichment fold means to what extent is the rate that co-occurring concepts from each setting actually appear in the Semantic Medline more than the average rate of all possible concept pairs in the Semantic Medline. Higher enrichment fold indicates higher possibility that the co-occurring concepts from each setting occur in the Semantic Medline more frequently than the average co-occurring rate in the Semantic Medline. Enrichment analysis for co-occurring concepts was performed in the SemMedDB using the Eqs. 2 and 3 to ultimately obtain the Enrichment Fold (Eq. 4).

2 $$ \mathrm{ProbExpSet}=\frac{\mathrm{TotalSemOcc}}{\mathrm{TotalPairNum}} $$

where TotalPairNum refers to the total number of possible concept pairs (e.g., (C0011849, C0015967)), among the concept collection from all randomly selected notes, TotalSemOcc refers to the total co-occurrence in the SemMedDM of all concept pairs from the concept collection. ProbExpSet calculates the average co-occurrence in the SemMedDB of all concept pairs from the concept collection, i.e., the expected probability for co-occurrence of concepts from each setting.

3 $$ \mathrm{ProbObsSet}=\frac{\mathrm{TotalSemOccSet}}{\mathrm{TotalPairNumSet}} $$

where TotalPairNumSet refers to the total number of possible concept pairs in each setting, TotalSemOccSet refers to the total co-occurrence in the SemMedDB of all concept pairs from this setting. ProbObsSet calculates the average co-occurrence in the SemMedDB of all concept pairs from each setting, i.e., the observed probability for co-occurrence of concepts from each setting.

4 $$ EnrichFold=\frac{\mathrm{ProbObsSet}}{\mathrm{ProbExpSet}} $$

The representative concepts for each setting was filtered with a threshold of enrichment fold over one.

### Topic modeling

In order to investigate the associations between problem list and practice setting, probabilistic topic modeling could serve as an effective method. Topic modeling has been useful to discover high-level knowledge and a broad range of themes from large collections of text documents. In biomedical domain, it has been applied in various aspects, such as discovering relevant clinical concepts and relations between patients [37], mining treatment patterns in Traditional Chinese Medicine (TCM) clinical cases [38], revealing clinical risk stratification from a large volume of electronic health records [39], clustering long-term biomedical time series such as electrocardiography (ECG) and electroencephalography (EEG) signals [40]. As a type of topic modeling, Latent Dirichlet Allocations (LDA) [19] has gained popularity in diverse fields since it holds great promise as a means of gleaning actionable insight from the text or image datasets. Howes et al. applied unsupervised LDA to analyze clinical dialogues as a higher-level measure of content [41]. Wang et al. developed BioLDA for the application in complex biological relationships in recent PubMed articles [42]. Flaherty et al. rank gene-drug relationships in biomedical literatures based on the LDA [43]. Chen et al. extended LDA by including background distribution to study microbial samples [44]. All these studies amplified the usability of topic modeling and LDA in biomedical field.

In this study, R package “topicmodels” [45] was used to build topic models for both setting similarity calculation and prediction purposes. Instead of using existing evaluation metrics [46–49], we chose the optimal number of topics in our data using log likelihood [50–52]. We calculated the log likelihood values with the number of topics varied from 5 to 150 by 5, and then investigated the performance by comparing the log likelihood value, of which the highest indicates the optimal number of topics. Additional file 1 shows the result of log likelihood method for choosing the optimal number of topics.

Then we fit an LDA model with the optimal number of topics using Gibbs sampling with a burn-in of 1000 iterations. To obtain the posteriors in the LDA analysis, we used collapsed Gibbs sampling because of relatively large number of topics in our study [53]. After we obtained the posteriors, we calculated the log-likelihood of the whole collection of problem settings by integrating all the latent variables.

To obtain setting similarity, the topic modeling was built first using all randomly sampled data, i.e., 1000 notes with chosen representative concepts per each setting, then setting topic probability of training sets was calculated based on the term topic probability derived from the topic models, specifically term topic probability associated with specific setting identified through representative concepts (terms) was extracted to calculate the average topic probability related to each setting. Pearson correlation coefficients among settings were calculated based on topic probabilities in settings using R3.2.1. Clustered Image Maps was then generated for visualization. Clustered Image Maps (i.e., heat maps) represent “high-dimensional” data sets by clustering of the axes to bring similar things together to create patterns of color [18]. To assess relationships between settings and problems, we generated clustered image maps [20] by: i) forming a matrix of the Pearson correlation coefficient among settings from all randomly sampled data, ii) clustering rows and columns of the resulting matrix, and iii) quantile-color coding of the resulting matrix.

To predict settings, all randomly sampled data was divided into 5 parts. Each part in turn was used to evaluate the settings derived by analysis of the other four parts, in the usual n-fold cross-validation manner. Setting prediction was conducted as follows:

- Setting topic probability of training sets was calculated based on the term topic probability for each setting derived from the topic models.
- Test data were predicted using the posterior function of the topic model derived from corresponding training data to obtain the setting topic probability using predicted term topic probability.
- Based on the setting topic probability, similarity was calculated among settings from training data and every one setting from testing data iteratively, so as to get the ranking order of the predicted settings based on Pearson correlation coefficient.

### Evaluation

Predicted settings for each tested setting were ranked according to their similarity. In order to evaluate the predicted performance, precision@k and recall@k (k = 2, 4, 6, 8, 10) were used for evaluation (Eqs. 5 and 6). For example, TP@k was calculated as the number of correctly predicted settings from top 1 to top k. FP@k was the unique number of correctly predicted settings from top 1 and top k reducing TP@k, FN@k was the total gold standard setting number (64) reducing TP@k. Based on the precision@k and recall@k, F1@k has also been derived (Eq. 7). We conducted a 5-fold cross validation, mean values were taken as the final evaluation results.

5 $$ \mathrm{Precision}@\mathrm{k}=\frac{\mathrm{TP}@\mathrm{k}}{\mathrm{TP}@\mathrm{k}+\mathrm{FP}@\mathrm{k}} $$

6 $$ \mathrm{Recall}@\mathrm{k}=\frac{\mathrm{TP}@\mathrm{k}}{\mathrm{TP}@\mathrm{k}+\mathrm{FN}@\mathrm{k}} $$

7 $$ \mathrm{F}1@\mathrm{k}=\frac{2^{\ast}\mathrm{TP}@\mathrm{k}}{2^{\ast}\mathrm{TP}@\mathrm{k}+\mathrm{FP}@\mathrm{k}+\mathrm{FN}@\mathrm{k}} $$

## Results

There were 3.3 million notes containing problems in an itemized format with a total of 18.9 million phrases or short sentences that are mapped to 4701 unique problem concepts. There were a total of 1265 settings out of which 266 were aggregated into 64 settings, consisting of 2.4 million notes (73% of normalized notes), and 113 thousand patients with 4573 normalized problems.

Results showed that enrichment folds are between 2.1 and 19.2 after TF-IDF and χ^2^ screening. As mentioned before, the threshold of enrichment fold more than 1 was used to filter representative concepts for each setting. These results indicated all concept pairs in each setting from randomly selected notes are significantly co-occurring in the Semantic Medline. We then used these concepts as the representative concepts for each practice setting.

**Fig. 2:**
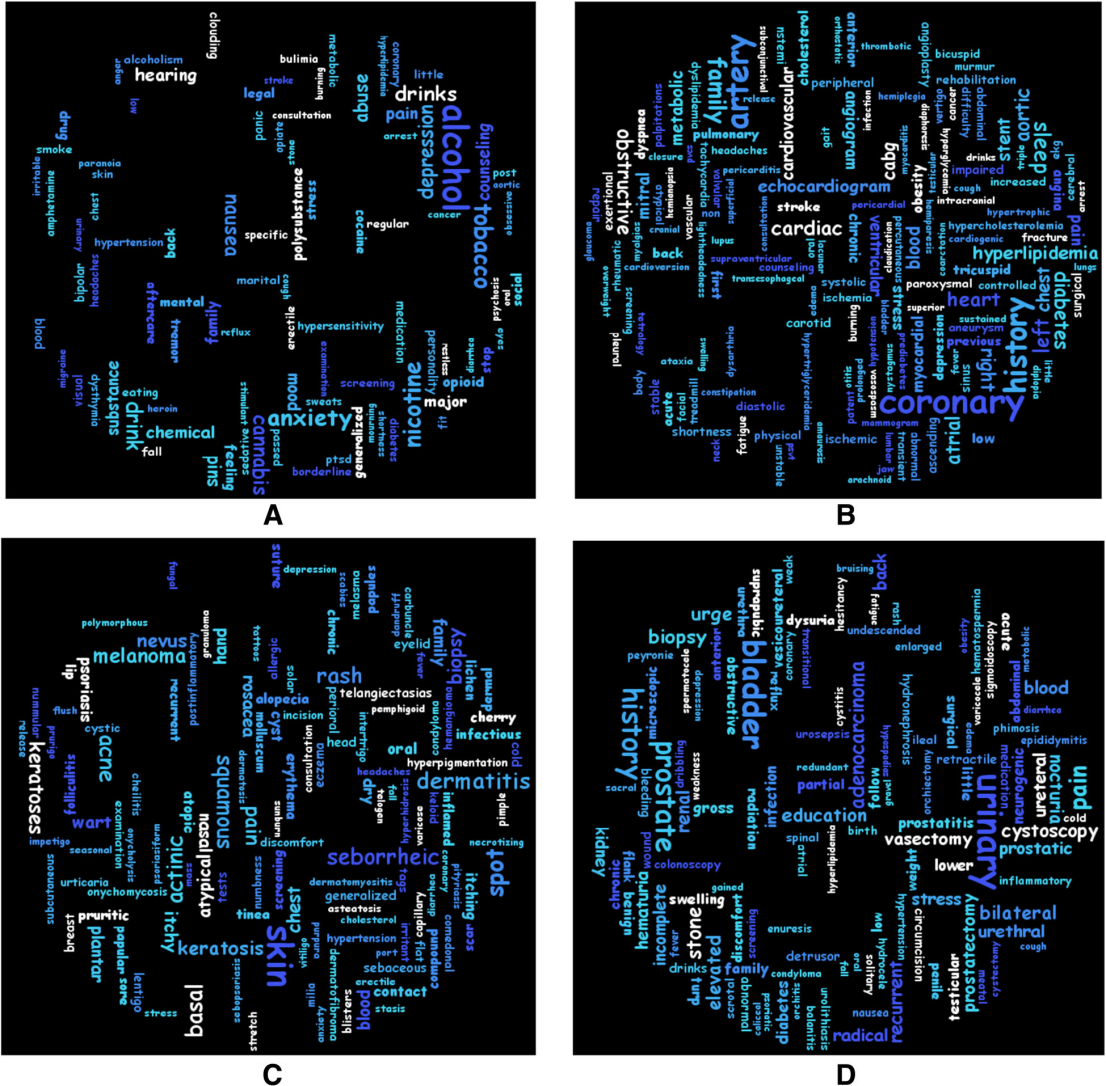
Concepts in various practice settings. **a**. Addiction setting. **b**. Cardiovascular setting. **c**. Dermatology setting. **d**. Urology setting. Font and color are assigned randomly, and font size is proportional to their significance, i.e., larger font means higher frequency of the term

Figure 2 is a word cloud figure developed using the open source software Kumo [54]. It showed the representative concepts from randomly selected four settings where larger font size means higher frequency. These concepts revealed the major themes of corresponding settings. For example, Addiction setting is featured by e.g., alcohol and nicotine, Cardiovascular setting by e.g., coronary and artery, Dermatology setting by e.g., skin and rash, while Urology setting by, e.g., urinary and bladder. Additional file 2 showed the frequency of top 10 concepts for each setting.

**Fig. 3:**
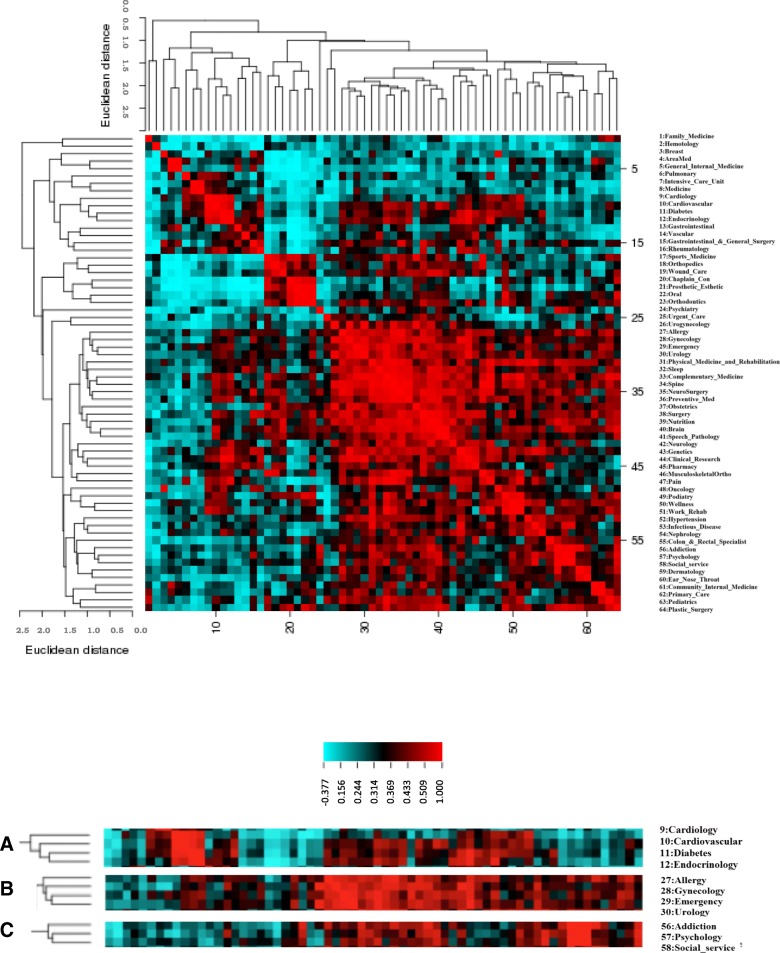
Clustered image map (CIM) of settings based on Pearson correlation coefficients (X-axis and Y-axis both are settings). Red color indicates positive correlation among settings, and blue color indicates negative correlation among settings. Figure 3**a**, **b** and **c** are three enlarged clusters.

Figure 3 showed the clustered image map [18] where a positive correlation (red color) indicates that problems in one setting or group of settings are similar to those in another setting or another group and a negative correlation (blue color) indicates that problems in one setting or group are different from those in another setting or group. From Fig. 3, we can see that some settings are highly similar, for example, Cardiology setting is similar to the Cardiovascular, Diabetes and Endocrinology settings (Fig. 3a). Allergy is similar to Gynecology, Emergency and Urology settings (Fig. 3b). Addiction is similar to Pychology and Social service settings (Fig. 3c).

Recall@k, Precision@k and F1@k were shown in Table 1. As k increases, the performance increases gradually. The reason that recall, precision and F1 are the same values when K equals 8 and 10 is FP equals FN when K increase to 8 and 10.

**Table 1:** Recall@k, Precision@k and F1@k (k = 2, 4, 6, 8, 10) for Pearson correlation coefficient

	@2	@4	@6	@8	@10
Recall	0.719	0.844	0.913	0.916	0.931
Precision	0.882	0.910	0.910	0.916	0.931
F1	0.790	0.875	0.911	0.916	0.931

## Discussion

During aggregating settings in Mayo Clinics, we have encountered the complexity in organization of the setting concept as stated in the study [29]. Due to the refined feature of practice settings at Mayo Clinic and for the purpose of simpler analysis, we have not totally aligned the extended setting values in Document Ontology (DO). First, we kept the settings that are similar but not exactly same, for example, Cardiology and Cardiovascular as separated settings. In contrast, in the proposed extensions to the DO [29] all settings are distinct. Second, we only used the extended setting values in DO in parallel, and have not studied settings in the hierarchy scenario [29]. For example, Emergency Setting is in parallel to Dermatology Setting in our study. While in the proposed extensions to the DO, Emergency Department is in parallel to Outpatient Setting that includes sub-level Clinic (Non-Acute) Settings, which embody the Dermatology Setting. Our mapping strategy kept features of clinical practices, and it could be used for future document hierarchy management.

In the clinical scenario, it is not easy for physicians from a specific setting to see the big picture with respect to problems most related to the setting. With the association between the problem list and practice setting revealed in our study, a prioritized and meaningful problem list above the irrelevant details could be generated, so as to help practitioners identify the most related problems from a succinct view. Our findings can predict practice setting based on problem list and providing a foundation for future document management. Furthermore, such findings also provide the premise for our next step toward automatic reformulation of problem lists as patients move from one practice setting to another, which would be a huge benefit. For example, when a patient is pursing help from the urology practice setting, his/her problems as the representative concepts associated with this setting, such as “bladder stones”, or “prostatitis” could be generated and presented to the physicians. When the patient moves to other practice settings such as cardiovascular practice setting, physicians can easily find the most relevant problems, such as “atypical chest pain” or “coronary vasospasm”.

From the practice setting level, highly associated settings which are unknown before can be revealed by using the similarity of problem lists. As shown in Fig. 3, Allergy is associated with Gynecology, Emergency and Urology settings. This finding will have implication in terms of health care for patients.

The reasons that we adopted LDA in our study instead of other methods include: 1) LDA is a unique bi-clustering approach with mixture models [55], considering both document-level and term level similarity. Other clustering methods such as k-means, can only cluster targets based on one similarity measurement. 2) LDA is also a robust generative Bayesian modeling approach, which specifically fits the big data analysis. The robustness is partially because LDA adopts conjugate distribution, such as Dirichlet and multinomial to build models. These features are unique in LDA which are not seen in many other unsupervised methods.

## Conclusion

To our best knowledge, our study is the first attempting to discover the association between the problem list and hospital practice settings. The contributions of our method are multiple. First, the NLP techniques normalizing problems from various settings enabled LDA analysis. With our negation function in NLP method, this analysis would be more accurate, compared with other studies [56]. Second, Semantic Medline was used for enrichment analysis of concept pairs to help identify representative concepts for each setting before feeding into LDA model. Third, setting similarity was visualized providing the general view among various settings. Forth, our method realized good prediction for practice settings using the similarity of topics derived from unsupervised LDA model, with the advantage of potential semantic associations among problems in settings. In the future, we plan to investigate how to provide more tailored care by utilizing the association between problem list and practice setting revealed in this study.

## Additional files

Additional file 1: Log likelihood values vs. Number of Topics. Note: The optimal number of topics is chosen when the maximum log-likelihoods are observed. This result includes a table showing the result of log likelihood method for choosing the optimal number of topics. (DOCX 100 kb)

Additional file 2: Frequency of concepts in randomly selected practice settings. This file includes a figure showing frequency information of top 10 concepts in each practice setting. (DOCX 13 kb)
