# Supplementary material for: Discovering associations between problem list and practice setting
Source: BMC Med Inform Decis Mak. 2019 Apr 4;19(Suppl 3):69. doi: 10.1186/s12911-019-0779-y (PMC6448189; doi:10.1186/s12911-019-0779-y)
Supplement: Supplementary file 1 — Log likelihood values vs. Number of Topics. Note: The optimal number of topics is chosen when the maximum log-likelihoods are observed. This result includes a table showing the result of log likelihood method for choosing the optimal number of topics. (DOCX 100 kb) [file 12911_2019_779_MOESM1_ESM.docx]

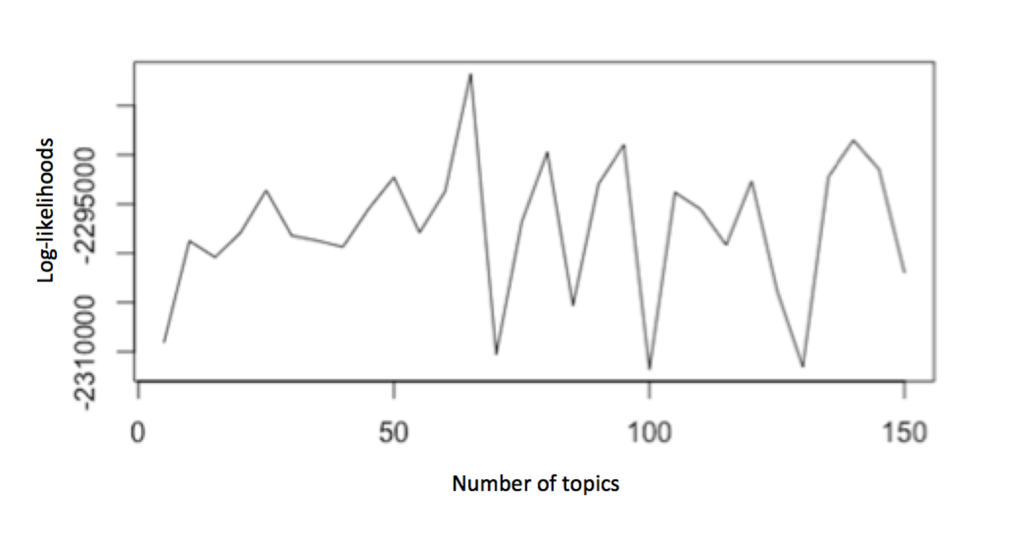


**Figure 1.  Log likelihood values vs. Number of Topics.** Note: The optimal number of topics is chosen when the maximum log-likelihoods are observed.
