# Supplementary material for: Discovering associations between problem list and practice setting
Source: BMC Med Inform Decis Mak. 2019 Apr 4;19(Suppl 3):69. doi: 10.1186/s12911-019-0779-y (PMC6448189; doi:10.1186/s12911-019-0779-y)
Supplement: Supplementary file 2 — Frequency of concepts in randomly selected practice settings. This file includes a figure showing frequency information of top 10 concepts in each practice setting. (DOCX 13 kb) [file 12911_2019_779_MOESM2_ESM.docx]

**Table 1. Frequency of concepts in randomly selected practice settings**

| **Addiction Setting**  **(Concept, Frequency)** | **Cardiovascular Setting**  **(Concept, Frequency)** | **Dermatology Setting**  **(Concept, Frequency)** | **Urology Setting**  **(Concept, Frequency)** |
| --- | --- | --- | --- |
| Anxiety, 418 | Coronary, 3217 | Skin, 754 | Pain, 250 |
| Drinks, 319 | Family, 980 | Spot, 267 | Erectile, 150 |
| Depression, 275 | Hypertension, 641 | Dermatitis, 212 | Biopsy, 123 |
| Pain, 130 | Diabetes, 576 | Acne, 204 | Recurrent, 115 |
| Family, 96 | Blood, 468 | Actinic, 197 | Blood, 84 |
| Chronic, 65 | Chest, 457 | Basal, 192 | Stress, 81 |
| Generalized, 49 | Pain, 388 | Seborrheic, 189 | Diabetes, 55 |
| Stress, 47 | Chronic, 217 | Rash, 181 | Family, 45 |
| Blood, 37 | Stress, 214 | Squamous, 181 | Chronic, 41 |
| Erectile, 25 | Cholesterol, 196 | Pain, 155 | Consultation, 36 |
